# Author's Reply

**DOI:** 10.1371/journal.pmed.0030167

**Published:** 2006-03-28

**Authors:** Karmela Krleža-Jerić

**Affiliations:** **1**Canadian Institutes of Health ResearchOttawa, OntarioCanada

It is a great pleasure to read that GlaxoSmithKline (GSK) is committed to registering its trial protocols and results [ 1]. I am also happy to hear that GSK intends to disclose at least the 20 items of the World Health Organization (WHO) minimal dataset, and does not believe there is a need for escrow. What a great example!

However, GSK's own registry is not what I would describe as an unbiased registry. It is a good and convenient tool to have—for GSK—but trials should be registered in a neutral, unbiased registry such as ClinicalsTrials.gov, the International Standard Randomized Controlled Trial Number (ISRCTN) Registry, or the Australian Clinical Trials Registry (ACTR).

Such registration will be a great step forward, as previously GSK and many other companies have often provided incomplete information to trial registries, when registering their trials. Following personal communication from D. Zarin at the time of the publication of my paper in *PLoS Medicine* [ 2], the National Institutes of Health (NIH) performed further analysis, and a paper was published in the *New England Journal of Medicine* [ 3]. This paper illustrates that even now—and despite medical journal editors' statements, WHO Guidelines, as well as other pressures such as the Ottawa Statement—industry inputs into ClinicalTrials.gov, which is the most often used trial registry, although improved, still leave much to be desired.

I disagree with Rockhold–Krall's statement regarding results versus protocol. Both are needed to ensure awareness of the relevant information for informed clinical decision making. I limited myself to protocol registration, because this is the first step that we need to take, and it has been much discussed lately. Without properly registered protocol information, we do not know the extent to which all the planned outcomes are reported or the nature and quality of the trial.

In other words, if we focus on results registration without knowing what was really studied, and thus not knowing which results were not reported, we are in an environment of outcome reporting or even of publication bias.

In the preregistration era, results were published, but as there was no up-front (prospective) trial registration, there was no way of knowing the real quality of such publications. Such up-front (prospective) protocol registration will enable the validation of the completeness of results reporting, which in turn will save a lot of sponsors' resources, particularly those of the pharmaceutical industry, and many lives. I would hesitate to participate, either as a trialist or as a study participant, in a trial that had not publicly disclosed at least 20 items. I would hesitate even more to prescribe or to use a drug developed through such a nontransparent process. However, I would be willing to consider prescribing and using a drug if I had a chance to analyse its potential risks and benefits. That would not have been possible without trial registration as we would continue to rely on the evidence based upon published results only, without knowing the scope of their accuracy

As of February 2006, we can say that we have defined, at a global level, a minimum-required protocol dataset via WHO's trial registration project ( http://www.who.int/ictrp). We shall now move on to registration and public disclosure of results. Of course, at the same time, we shall continue registering trials in member registries that provide at least 20 items. This must be done globally.

The lessons learned in building the culture of registration will be used to improve the quality of results reporting. Had we moved on to results too soon, it would have distracted us from pinning down the essential protocol information, which would have opened the window for continuing manipulation of results presentations.

Furthermore, from a public health perspective, it is my hope that we shall revisit and further develop the minimal protocol dataset once we have been through the exercise of defining the registration of the results. This is why the Ottawa Group is continuing a dialogue beyond the minimal dataset.

I have no illusions that all data for all trials will be registered, right now, but we have made a good start in requesting a minimal 20-item dataset. I agree with Rockhold and Krall that we shall have to see how many of these 20 will be kept secret.

Discovery and development in medicine is, indeed, a very important activity for society, and the pharmaceutical industry plays an important role. With regard to the social contract between industry and society, let me point out that social contracts are based upon mutual respect and confidence, and they are open to revisions. Trial registration—as a tool of transparency, knowledge sharing, and accountability—will help restore the confidence of society in the pharmaceutical industry, which is currently very fragile, and thus enable the (re-)establishment of a (new) social contract between these two parties.

I have looked again at the continually growing list of endorsements of the Ottawa Statement (OS) Part 1 [ 4] (available at http://ottawagroup/ohri/ca), and there is still no industry endorsement. Neither Rockhold nor Krall has signed the OS1, not even after their letter. Since they have declared they are “all for registering”, I am inviting them both again to sign the OS1, and to comment and consider endorsing the OS2 on principles of implementation [ 4].
