# Correction: Author's Reply

**DOI:** 10.1371/journal.pmed.0030251

**Published:** 2006-05-30

**Authors:** Karmela Krleža-Jerić

In *PLoS Medicine*, volume 3, issue 3: DOI: 10.1371/journal.pmed.0030167

The URL provided for the Ottawa Group was incorrect. It should be http://ottawagroup.ohri.ca/signatories.html.

